# Severe Cholestatic Hepatitis due to Temozolomide: An Adverse Drug Effect to Keep in Mind. Case Report and Review of Literature

**DOI:** 10.1097/MD.0000000000000476

**Published:** 2015-03-27

**Authors:** Antonio Grieco, Maria Antonietta Tafuri, Marco Biolato, Barbara Diletto, Nicola Di Napoli, Nicola Balducci, Fabio Maria Vecchio, Luca Miele

**Affiliations:** From the Institute of Internal Medicine, Catholic University of Rome (AG, MB, LM); Institute of Radiotherapy, Catholic University of Rome (MAT, BD, NDN, NB); and Institute of Pathology, Catholic University of Rome (FMV).

## Abstract

Temozolomide is the current standard of therapy for postoperative patients with glioblastoma starting adjuvant radiotherapy. Hematologic adverse events are the most frequent side effects of temozolomide, while liver toxicity has been reported only in the post-marketing period.

Here we report a case of severe temozolomide-induced liver injury during concurrent radiotherapy treatment, at a dose level of 75 mg/m^2^.

The aim of this case report is to focus on the problems of temozolomide-induced hepatotoxicity. In conclusion, a close monitoring of liver function tests is recommended during treatment with temozolomide.

## INTRODUCTION

Currently first-line adjuvant treatment of glioblastoma is based on the association of radiotherapy and temozolomide, an oral alkylating agent.^1^ Combination treatment has been demonstrated to significantly improve 5-year survival and progression-free survival compared to radiotherapy alone.^2^ Combination therapy, however, increases the rate of side effects. In particular, temozolomide has been associated with anemia, lymphopenia, neutropenia, and severe thrombocytopenia.^1,3–5^ Temozolomide-induced liver injury represents a better defined problem in a monotherapy (postradiotherapy) high-dose treatment regimen, where dose levels range from 150 to 200 mg/m^2^, while during concomitant treatment with radiotherapy the dose level is 75 mg/m^2^. In the latter setting, temozolomide-associated hepatotoxicity is still undefined.^4,6–8^

## CASE REPORT

**FIGURE 1:**
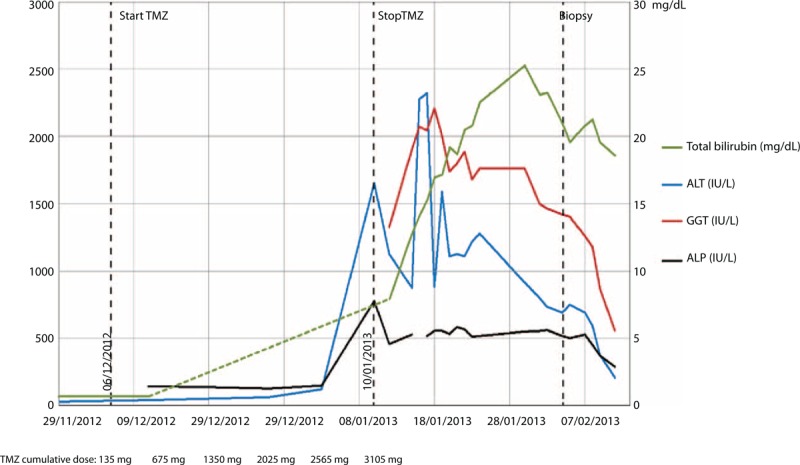
Liver function tests of the patient during the treatment course.

**FIGURE 2:**
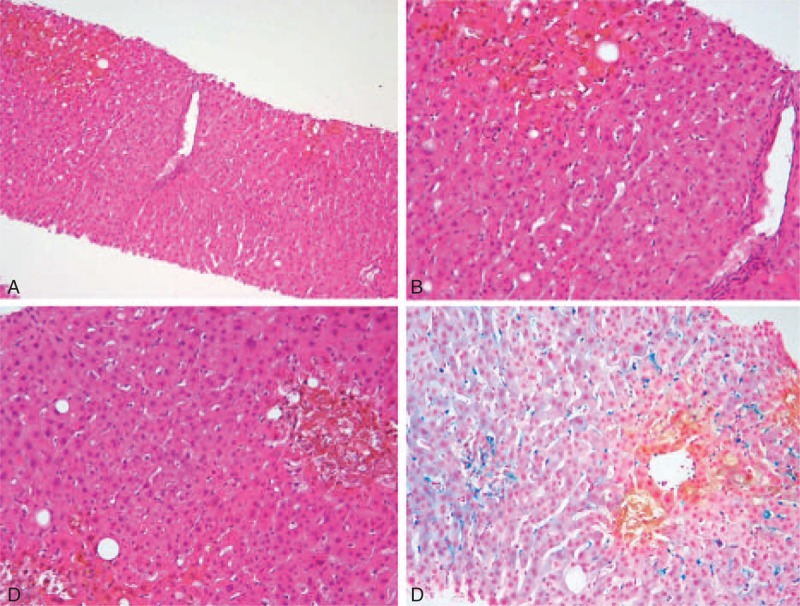
Liver histology. A and B: Portal tract (in the middle) and centrilobular cholestasis [10× (A), 20× (B) original magnification]. C and D: Mild steatosis and Kupffer cell activation with hemosiderosis are associated with zone 3 cholestasis (C: 20 × original magnification, D: Perls stain).

A 67-year-old man with no previous medical history was admitted in neurology clinic at “Gemelli” Hospital on May 2012 because of gait disturbance and face perceptual deficit (prosopagnosia). Cranial gadolinium– enhanced magnetic resonance imaging showed a lesion suspected to be a primary malignant brain tumor in the right temporo-parietal-occipital lobe. The patient underwent right fronto-temporal-parietal craniotomy and excision of the lesion. In adherence to the neurosurgery protocol, he started postoperative antiedema treatment with dexamethasone (4 mg twice daily) and esomeprazole (40 mg once a day). The histological report confirmed the diagnosis of glioblastoma multiforme. On day 41 after resection the patient started adjuvant combined chemoradiotherapy, based on 60 Gy (2 Gy per fraction) with concomitant oral temozolomide 75 mg/m^2^/day according to current guidelines.^1^ Baseline laboratory workup was unremarkable with normal blood cell count and normal liver function tests. One month later, the patient was hospitalized due to severe asthenia and jaundice. On admission, laboratory test results showed severe cholestatic hepatitis: alanine aminotransferase (ALT) 1128 IU/L; serum total bilirubin 7,96 mg/dL; conjugated bilirubin 5.75 mg/dL; γ-glutamyltransferase (GGT) 1325 IU/L; alkaline phosphatase 458 IU/L; prothrombin time 98%, platelets 202 × 10^9^/L, WBC 10.79 × 10^9^/L, creatinine 0.78 mg/dL. Viral (hepatitis B, hepatitis C, hepatitis A, cytomegalovirus, Epstein–Barr virus, rubella virus, herpes virus) and autoimmune etiologies were excluded. Abdominal ultrasound and computed tomography examination did not reveal pathological findings. A chart of the liver function test levels is reported in Figure 1. Temozolomide was suspended. The patient started ursodeoxycholic acid 300 mg TID po, ademetionine 400 mg BID i.v., methylprednisolone 40 mg QD i.v., and multielectrolyte solution in 5% dextrose 2000 mL QD i.v. Unfortunately, liver function did not improve and total bilirubin level rose to 25.28 mg/dL, ALT to 2322 UI/L, GGT to 2074 UI/L, and alkaline phosphatase to 780 IU/L. Liver biopsy was performed. Histological examination showed severe centrilobular and canalicular cholestasis with preserved hepatic architecture. The findings were considered consistent with toxic liver injury (Figure 2). Three weeks after admission, despite slow progressive improvement of all liver function tests, the patient's general conditions worsened and he died of *Staphylococcus aureus* sepsis 30 days after admission.

## DISCUSSION

Here, we report a case of severe cholestatic hepatitis occurring in a patient affected by glioblastoma and treated with temozolomide at the standard dose. According to current practical guidelines for diagnosis and management of drug-induced liver injury,^9^ the absence of previous liver disease and the absence of other drugs in temporal relationship with the start of therapy point to temozolomide as the probable cause of the observed liver injury. The liver toxicity seems to be of idiosyncratic type. Dixit et al in their systematic review of temozolomide-related toxicity collected five cases of liver injury with a clinic-pathological picture of cholestatic type in four cases^5^; the author reported a median cumulative dose of 3075 mg and a median latency of 6.5 weeks from start of temozolomide. Our patient fits Dixit's series well: symptoms appeared 4 weeks after the start of therapy after a cumulative dose of 3105 mg. Currently, temozolomide tolerability represents one of the main concerns for its use. Niewald et al, in a retrospective study of 46 patients treated with simultaneous radiochemotherapy with temozolomide, reported two cases of significant elevation of liver function tests leading to interruption of chemotherapy: In one of these cases a hepatitis B reactivation was observed.^4^ It is still unclear whether a longer duration of adjuvant therapy with temozolomide may influence the rate of relapse. Even when administered in an intensified regimen, temozolomide is documented to have negligible nonhematological toxicity. Recently, Weiler reported only one case of hepatitis and one case of subclinical liver enzyme elevation among 41 patients treated.^3^ The study by the Manitoba Cancer Center with 116 patients treated with adjuvant temozolomide and *cis*-retinoic acid for up to 24 cycles or until evidence of disease progression documented 13 patients with elevated transaminases and three cases of elevated bilirubin, but none of these patients discontinued therapy.^10^ Our case closely resembles the lethal case of cholestatic hepatitis reported by Sarganas in relation to type of liver injury and the absence of previous liver pathology,^7^ but differs regarding the time of onset of liver toxicity: While Sarganas reported the appearance of symptoms at the end of therapy, we observed the start of transaminase elevation after 3 weeks of therapy. The authors reviewed data from the Food and Drug Administration (FDA) adverse event reporting system and recognized seven cases of temozolomide-induced liver injury, but in two cases other drugs potentially responsible for liver toxicity were involved. Among the other reported cases, only the case described by Goldbecker seems to resemble ours because of the absence of other drugs potentially involved and the latency period of about 2 months.^8^ The role of drug-to-drug interaction must be further discussed, as also the potential interaction with complementary and alternative medicines, as recently reported.^11^ In the cases reported in the literature, concomitant administration of valproic acid or pantoprazole has been described: but the role of P450-dependent metabolism seems to be marginal.^7^ Our patient was not taking drugs or complementary and alternative medicines other than temozolomide.

In December 2013, the European Medicines Agency on the basis of 44 cases of severe liver injury (including some fatal cases) reported in the post-marketing period published an informative notice on temozolomide.^12^ According to this recommendation, liver function tests must be carried out at baseline, at midcycle, and after each cycle of treatment (each cycle of treatment lasts 42 days).

In conclusion, the importance of the role of temozolomide in the treatment of glioblastoma makes the correct evaluation of side effects, including liver injury, crucial. Before and during treatment with temozolomide, monitoring of liver function tests is recommended according to the schedule proposed by the European Medicines Agency.
